# Bilateral hilar lymphadenopathy in a young female: a case report

**DOI:** 10.1186/1752-1947-1-60

**Published:** 2007-08-03

**Authors:** Seema Varma, Shilpi Gupta, Raymond ElSoueidi, Meekoo Dhar, Jotica Talwar, Neville Mobarakai

**Affiliations:** 1 Division of Hematology and Oncology, Department of Medicine, Sanford R. Nalitt Institute of Cancer and Blood Related Diseases, Staten Island University Hospital, 256 Mason Avenue, Staten Island, New York, 10305, USA; 2 Department of Pathology, Staten Island University Hospital, 475 Seaview Avenue, Staten Island, New York, 10305, USA; 3 Division of Infectious Diseases, Department of Medicine, Staten Island University Hospital, 475 Seaview Avenue, Staten Island, New York, 10305, USA

## Abstract

Hilar or mediastinal lymphadenopathy is not included in the wide spectrum of radiologic findings associated with bronchiolitis obliterans-organizing pneumonia (BOOP). We present a patient who presented with extensive hilar and mediastinal lymphadenopathy. We suspected a diagnosis of sarcoidosis. The patient was diagnosed with idiopathic BOOP. This is the first case demonstrating that BOOP, now referred to as cryptogenic organizing pneumonia (COP), can present with bilateral hilar lymphadenopathy.

## Background

We present the case of a young woman with presentation suggestive of sarcoidosis. She had extensive hilar and mediastinal lymphadenopathy that directed the differential diagnosis and further work-up.

## Case presentation

A 37-year-old African American woman with past history of hypertension on no medications who migrated to USA from Jamaica 5 years ago presented with persistent dry cough, intermittent low-grade fever, night sweats, fatigue, weakness and dyspnea of exertion of 6 weeks duration. There was no history of orthopnea, paroxysmal nocturnal dyspnea, exposure to toxic gas or organic dust, loss of weight or appetite, fever and joint pain. She was a non-smoker and social drinker.

**Figure 1:**
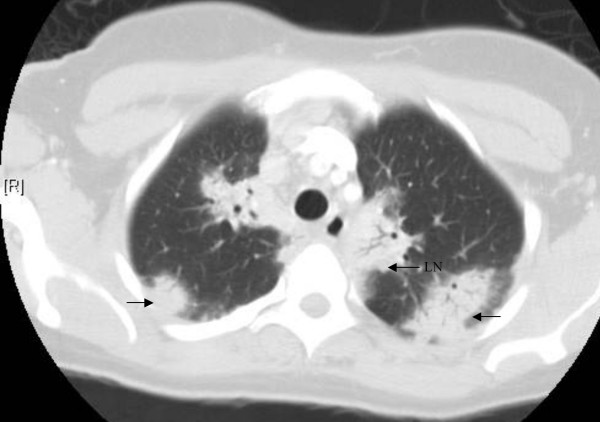
CT scan of the chest revealing peripheral consolidations and perihilar consolidations with hilar and mediastinal lymphadenopathy.

On admission, temperature was 100.2°F; pulse, 113 beats/min; respirations 18 breaths/min; and blood pressure, 150/80 mm of Hg. The partial pressure of oxygen was 60 mm of Hg on room air. Rest of her physical examination was normal. Laboratory data showed: white cell count, 11,600 cells/μL, with 82% granulocytes and 13% lymphocytes; hemoglobin, 11.6 g/dl and mean corpuscular volume 82 femtoliters; platelet count, 518,000 cells/μL; erythrocyte sedimentation rate 117 mm/hr and C reactive protein 7 mg/dl. A chest radiograph showed nodular infiltrates in bilateralupper lobes of the lungs and peri-hilar fullness. CT scan showed extensive bilateral hilar and mediastinal lymphadenopathy with areas of perihilar and peripheral consolidation (Figure 1). Pulmonary function tests demonstrated a mild restrictive pattern.

Differential diagnosis included atypical pneumonia, tuberculosis, fungal or other opportunistic infections, sarcoidosis, interstitial lung disease, connective tissue and autoimmune disease, lymphoma or occult malignancy. The patient did not respond to an antibiotic regimen of erythromycin and ceftriaxone that was later changed to moxifloxacin. Initial as well as repeated blood and sputum cultures for bacteria, mycobacterium and fungus were negative. PPD and HIV ELISA test were negative. Analyses for rheumatoid factor, anti-nuclear antibodies and antineutrophil cytoplasmic antibody that resulted at a later date were negative. CT scan of the abdomen and pelvis was negative.

A mediastinal lymph node biopsy showed only reactive anthracosis and no evidence of granuloma or malignant cells. Despite the negative biopsy results, sarcoidosis was still high on the differential considering the typical clinical presentation, typical radiologic findings and the age and descent of the patient.

**Figure 2:**
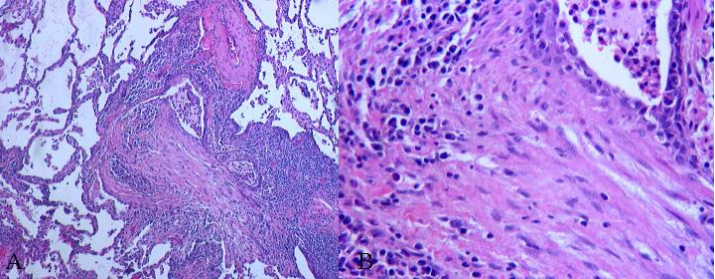
Photomicrograph of hematoxylin & eosin stained slide (low [A] and high [B] magnification views) showing patchy fibrosed areas, obliterated bronchiole and chronic inflammatory infiltrate with preserved lung architecture.

We finally proceeded to an open lung biopsy, which showed sharply demarcatedpatchy fibrosed areas with fibrotic plugs and lymphocytes, plasma cells, macrophages, neutrophils and foamy macrophages (Figure 2). This confirmed the diagnosis of Bronchiolitis obliterans organizing pneumonia (BOOP) [1]. Patient was startedon oral prednisone 1 mg/kg/day with dramatic improvement both clinically and radiologically in 8 weeks. The prednisone dose was gradually tapered and stopped after 12 months. During 1 year of follow-up, the patient has remained asymptomatic.

## Discussion

Typical histopathology and dramatic response to steroid therapy definitely favor the diagnosis of BOOP in this patient, however, the clinical and radiologic findings were highly suggestive of sarcoidosis. Clinically it may be difficult to differentiate BOOP from sarcoidosis. Clinical presentation can be similar for both. Radiologically, bilateral perihilar and peripheral consolidations can also be associated with both. Butpresence of extensive bilateral hilar and mediastinal lymphadenopathy has strongly been associated with sarcoidosisand has not been associated with BOOP.

BOOP, which was first described in 1985 [1], now more commonly referred to as cryptogenic organizing pneumonia (COP), can present with a wide variety of radiologic manifestations. A review of the literature revealed that presence of mediastinal lymphadenopathy on radiological imaging has rarely been associated with BOOP. A study conducted to determine prevalence of mediastinal lymphadenopathy in BOOP at University of British Columbia concluded that BOOP can be associated with enlarged mediastinal lymph nodes but usually not more than two lymph nodes are enlarged [2]. The patient we present had extensive mediastinal lymphadenopathy rarely seen in BOOP patients. Gupta et al [3] reported the only case of BOOP presenting with hilar lymphadenopathy. They explained the hilar lymphadenopathy on imaging studies as probably being pneumonic foci in hilar or peri-hilar location. Extensive bilateral mediastinal lymphadenopathy with bilateral hilar lymphadenopathy which is classic for sarcoidosis has not been reported with BOOP.

The etiology of BOOP remains unknown in majority of cases. Associated with sarcoidosis, BOOP has been described as a complication of lung transplantation in patients with end-stage pulmonary disease [4] and in association with alveolar sarcoidosis [5]. BOOP occurring independently mimicking the presentation of sarcoidosis has not been described.

Based on the negative work-up panel, typical histopathologic findings, no response to antibiotics, dramatic response to steroid therapy and present good health of the patient after cessation of therapy; we believe that our patient had idiopathic BOOP.

## Conclusion

This is the first case of BOOP presenting with extensive bilateral hilar and mediastinal lymphadenopathy. This case demonstrates that bronchiolitis obliterans-organizing pneumonia (BOOP), now referred to as cryptogenic organizing pneumonia (COP), can both clinically as well as radiologically mimic sarcoidosis. This entity must be included in the differential diagnosis of hilar and mediastinal lymphadenopathy.

## Abbreviations

BOOP – Bronchiolitis obliterans organizing pneumonia

COP – Cryptogenic organizing pneumonia

PPD – Partial protein derivative

HIV – Human Immunodeficiency virus

ELISA – Enzyme linked immunosorbent assay

## Competing interests

The author(s) declare that they have no competing interests.

## Authors' contributions

Seema Varma was involved in conception of the case report, data collection, review of literature and writing the manuscript. Shilpi Gupta and Raymond Elsoueidi participated in data collection. Jotica Talwar participated in pathologic diagnosis and data collection. Neville Mobarakai coordinated and helped to draft the manuscript. All authors read and approved the final manuscript.

## References

[B1] Epler GR Colby TV McLoud TC Carrington CB Gaensler EA Bronchiolitis obliterans organizing pneumonia N Engl J Med 1985 312 152 158 3965933 10.1056/NEJM198501173120304

[B2] Niimih H Kangey EY Kwong JS Carignan S Muller NL CT of chronic infiltrative lung disease: Prevalence of mediastinal lymphadenopathy J Comput Assist Tomogr 1996 20 305 308 10.1097/00004728-199603000-00026 8606243

[B3] Gupta PR Joshi N Khangarot S BOOP presenting as pseudolymphadenopathy Indian J Chest Dis Allied Sci 1999 41 235 240 10661013

[B4] Walker S Mikhail G Banner N Partridge J Khaghani A Burke M Yacoub M Medium term results of lung transplantation for end stage pulmonary sarcoidosis Thorax 1998 53 281 284 9741371 10.1136/thx.53.4.281 PMC1745184

[B5] Rodriguez E Lopez D Buges J Torres M Sarcoidosis-associated bronchiolitis obliterans organizing pneumonia Arch Intern Med 2001 161 2148 2149 10.1001/archinte.161.17.2148-a 11570948

